# Histology and Microscopy*From the published Transactions of the American Dental Association

**Published:** 1878-03

**Authors:** W. H. Atkinson


### THE DENTAL REGISTER.

VOL. XXXII.] **MARCH, 1878.** No. 3.

### HISTOLOGY AND MICROSCOPY, AN ESSAY*

BY W. H. ATKINSON, M. D., D. D. S.

This is a broad subject, deeply involved, complicated, profound, and occult; but beginning to take on scientific aspects.

Anything like a satisfactory account of the rise and progress of histology would involve a conspectus of the origin and career of the whole range of Natural History, Medicine, and Surgery. The study of these departments has brought to view whatever is known of histological or microscopic science.

The mere fact of our being in possession or not of the knowledge of science, art or commerce, neither creates nor annihilates any part, nor the whole, of the categories referred to. For they must be, to become catalogued as knowledge. Therefore the knowledge does not make or unmake any part of the field of contemplation in scientific or social order of life.

*From the published Transactions of the American Dental Association.

The so-called "exact sciences" become exact only by lodgment in the minds of those capable of comprehending the processes by which they are rendered regular and certain, or, in other words, by our becoming acquainted with the nature and career of physical and mental processes.

Histology can only be known through direct revealment to the consciousness, or indirectly through microscopical machinery. Hence the great propriety of coupling the two (histology and microscopy) in the caption of a paper setting forth whatever may be provable to the senses in the career of tissues and their elements in *associate* and *dissociate* presence.

This first glance at the subjuct startles us as we begin to take it into our consciousness in its comprehensive and dispersive aspects; for it really strikes at the foundations and superstructures of all knowledge of all kinds and degrees.

The Microscope is comparatively a modern invention. Therefore, there was no real knowledge of tissues before its discovery, invention, construction and employment; or, what was known came into consciousness through other channels. In fact, the possibilities and principles of the microscope had to find lodgment in the mind before it could be discovered, invented, made and used—each stage of the progress of the revealments of principles and possibilities, no less than the final summation, solution and applications to practice, requiring immediate influx. When we shall know what influx is, and be able to express it in understandable phrase, we shall have a basis of certitude in this department of science, as absolute as that known to pertain to the relations of number. All becoming, in physics and consciousness, depends upon afflux and efflux, and is only capable of cognition when complete. Just here is the great hindrance to equable and certain progress in attainment.

The recorded categories of past observations are so vague and embryonal, that they do not meet the mental appetite of the present with statement sufficiently strong, broad and clear to satisfy. All search for new and complete categories must proceed upon immediate influx, or recapitulations of former influxes, in reasoning upon the known to reach the unknown.

It will be profitable for us to ask what reasoning is, and to learn that it is a holding of the values of concealed processes in a sort of compressed essence in such a state as to polarize the mind so as to admit of a new influx (revelation) of the knowledge desired. Thus *sentiment* is inherited essence of logic, and by no means to be scouted or disregarded as non-essential to the attainment of truth.

Career of body and being, fact and function, in physics and consciousness, involves the questions of origin and destiny, or plan and purpose, in mode and manner of differentiation of occupancy of habitat.

Confluence and diffluence are the diastole and systole of career to the departments in which they occur.

Body is echo of body, as shadow is sensuous proof of substance—substance being the subtrate of spiritual and material forms of body—there being no possibility of body that does not partake of the dual primates of substance called spirit and matter inhering in it, in dynamic interdependence.

Histology to-day is crippled most in the house and hands of its own children and friends, by their persistence in carrying questions and methods of one category into another to which they are not adapted in nature, significance and use. For instance, it is still held to be important to "settle," as they say, the "priority" of "body or germ," "oak or acorn," "egg or hen." It is not "settled" what "germ" or "body" is. How then can "priority" of presence in any category be determined? Whether minutest granules shall be regarded as eggs, and what degree of ripeness of these shall constitute fitness for impregnation, or whether a sort of parthenogenesis hold genus, species and variety in its potential grasp, is not yet "settled" for all the forms already known. How infinitely less, then, can be "settled" the verities of the vastly more extended fields of the unknown bodies and career that now so vehemently press for scientific revealment in the study of a true histology, until we can cancel and cast behind all that stands in the way of solution of body and process?

When we depended for demonstration of elemental bodies upon natural vision, we could only go to tissues as elemental. But when the microscope was revealed, the present acknowledged elements of these then elemental bodies were revealed to sight, and at once became the objects of study and controversy. And we now stand on vantage ground, and are able to solve previously insoluble problems of presence, and modes of presence of body and process. What, then, ought we to be ready to cancel and cast behind? Nothing of value, only the embryonal and transitional—not the fruits of these in values of verity.

At one time, and for the time appropriately, the solidal pathology held sway. In due time the fluidal made its advent upon the arena of function, and had its day of domination; but of shorter duration, in consequence of increasing intelligence and attention, the continued exercise of which in turn made it give way to the advent of the cellular pathology, ushered in by the use of the microscope. And now this is about to give up its differential dominion in favor of the summation of all three in the concurrence of these in Pragmatic Pathology.

Pragmatic, or pronounced, pathology can only find lodgment in the minds of men by the study of body and process upon a plan so natural, general and definite, as to convince of its verity and applicability to the categories of function.

That every factor of function must be, before it can act, and act repeatedly in like manner to establish habit by which it may be known, must be apparent to all capable of thinking upon the subject.

The law of becoming, in body and function, is the law of succession or parentage.

Becoming can only be known by *revelation* to consciousness.

A seriating, then, of motion and modes of motion, is a necessity out of which categories may be evolved.

There are seven degrees or modes of motion regnant in the plan of individual being, small or great. And these may be nominated either way, but always in a regulated series down and up, or up and down.

Down.—1st, (Spirit) Power; 2d, (Vital) Force; 3d, Chemism; 4th, Electrism (including magnetism); 5th, Light; 6th, Levitation, and 7th, Gravitation.

Up.—1st, Gravitation; 2d, Levitation; 3d, Light; 4th, Electrism; 5th, Chemism; 6th, (Vital) Force, and 7th, (Spirit) Power.

The first or downward procession indicates what has been called "Creation." The second or upward procession is Procreative, Functional, Elaborative, or relegative (a seeking to return whence it came), and is the basis of *associative* and *dissociative* movements among the elements of tissues.

*Gravitation* holds the dominion of differential individuation. *Spirit* holds the dominion of indifferentiation, or sameness of all power and presence, and is the unitary cause of all motions and modes—therefore, the universal of Being in highest tension.

A striving between these two modes of tension produces Suns, Systems, Cycles, Circles, Planets and Inhabitants of Planets.

The tissues of our bodies hold the relations to the organs that the inhabitants hold to planets, and the organs hold the relations of circles and cycles to the system of organs that constitutes our individual bodies.

"Just as the study of the whole class of *Radiates* in general, and of jelly-fishes in particular, reveals the law, plan, type and methods of production (that so long defied proper classification, and now shows these methods of production and variation repeated and amplified in the higher and more complicated fields of geological research), and without an apprehension of which, persistently carried in the mind, can any zoologist make regular, rapid and profitable observations," even in the special department to which he may be devoted. Even so does the study of the seven modes of tension reveal cosmical and planetary law regnant everywhere among the largest and the least of individual—Cycle, Circle, System, Organ, Tissue, Cell and Molecule—wherever process is, actual, known or potential. In fact, until we apprehend that the whole is potentially, typically or analogically present in the least, or that the universe is this whole, whose presentment in departments capable of cognition presents us with the segregate modes of motion, or currents, in analogous, concurrent or competing presence, shall we be able to solve any problem in histology.

Becoming involves a deep and hidden or unseen current, or set of currents, that come from without, and appear within the range of sense.

This last word "sense" involves another realm upon which it depends, and without which it can not be, viz: the realm of consciousness, the basis of all knowing, or knowledge of being, body and process.

The confluence of consciousness and physics posits individual being or beings.

The primal example of individual being is an ideal body denominated the Atom.

Confluence of *atoms* produces *molecules*, and aggregations of these constitute what are nominated *granules*. This last is the first and least body capable of making its presence known to the senses, and therefore stands as daysman between the ideal and corporeal domain of bodily presence. Aggregations of granules, in regular order, produce functioning bodies called corpuscles, cells, etc., according to the fancy of him who catalogues the objects under consideration.

I said the strivings of differentiation (gravitation) and indifferentiation (spirit) produced Suns, Systems, Planets and Inhabitation of Planets.

A mode of striving known as affinity (kindred) produces the different forms of molecules, and their modification in granules, cells, tissues, organs and systems, under the tentions of afflux and efflux of currents of force, that make and unmake these bodies at the behest of type, plan, purpose.

Observation has taught us that "like produces like," and "unlike, diversity," in all the departments of individual and associate body. It also teaches the doctrine of cancellation of one form of body in the advent of another, in every example of production and maintenance of physical and functioning body. So that the debris of one grade of body becomes the food of the next to it, by reason of the differences of tension of force and form in the deficiencies or wants, and the sufficiencies or fullness, of the digestory receptacles of the bodies concerned. This teaches that state of depletion or repletion of body becomes the point of invitation to or repulsion of the currents of force that constitute the disintegrating and the integrating, or the destructive and constructive, processes of killing and chaotifying one body for the support of another—the *force* or *life* of which never dies, the form only dying, or rather changing by transmutation or metamorphosis into a more complicated or less complicated form in accordance to the surrounding influences within the sphere of the currents.

All the molecules, cells, tissues, organs and systems with which we are acquainted are but the channels of, and the results of, modes of motion, and they are mutually transmutable into each other, under the dominion of force, as certainly as that light, heat and electricity are modes or phases of motion —though more subtle, fine and evanescent than the bodies upon which we observe their effects—and are the occasion of the conversion of these forms of motion into each other.

So far as we yet see, by the best aids to vision, tissual elements arise in an apparently amorphous mass of what we now call Protoplasm (or first mould).

That which is called digestion, in the broad sense of feeding, consists in comminuting and solving the food, consisting of organs, tissues, cells and granules, into the amorphous molecular mass named Protoplasm, proteinaceous mass, chaotic mass, blood and pabulum, in which differentiation makes its advent, and does its work of producing the factors of differential function in Sensory, Motory, Secretory, and Psychic examples of function.

These are the phases of confluence, of consciousness and physics that lay at the bottom or base, and set up the demand for the varieties of tissues as elaborators of the various functions called for to complete the system of organs to which they belong.

We use these terms with clearness of meaning and propriety of significance, or with ambiguousness of meaning and indefiniteness of understanding, in accordance with the time in which we write or speak, and the stage of our education or evolution of the knowledge of process and body, as displayed in the study of histology as seen by the help of a rapidly advancing microscopy.

In fact the microscope has so extended the domain of sight, and cleared up and amplified the apprehensions of body, that we now prefer to investigate for ourselves rather than to read the accounts of the researches of others, as guides to a knowledge of functions, which is the objective point of all histological acquirement.

*Histos*, a web, and *logos*, a discourse, are the etymological primates from which we derive our word Histology.

Some webs are made of homogeneous sheet mass; others are woven of fibrous or thread like, or line like elements loosely or closely laid together to form the web or sheet.

Matter being indestructible, and force being persistent, they must be regarded as aspects of a something upon which they depend, which something we may call substance, of which the universe consists—which universe, as a whole, is a plenum, and therefore understandable from the comprehension of any one of its system within our reach for study.

What we call extremes of size are only so by way of accommodation of phraseology. For the molecule too small to be seen by the assistance of the highest microscopic amplification, is not an extreme but a mean of expressions of force and form, which mean raises it out of the range of mere atoms, and makes it no longer an elemental but a compound body, endowed with new and complicated affinities by which it may be related in manifold method and mode. On the other hand, the astronomical bodies, territories, and spaces have been also denominated extremes, only, again, by way of accommodation; for, as our knowledge increases, our extremes become only the mean of that which is ever ready to be and ever is revealed as but the mean between the known and the about to be known.

So, if we would solve the problem of the universe, we must understand the relations of the parts of systems to each other, and thus become acquainted with the analogies that unfold the laws of the whole. The only way that this can be done by one person is to take examples of systems of short career and small dimensions, and so transparent as to reveal the metamorphoses through which they pass from inception, ripeness and decadence, to disruption and dispersion into the elements whence they came.

Fortunately for us, many examples in the vegetable and animal world afford us such opportunity. And we may repeat our observations until the whole range of production, growth, death and dispersion is familiar to us.

This patient watching of the ripening of germs, their impregnation and evolution into adult form and fruiting form again is but the true work of the histologist.

The finest naturalists are to this day unable to distinguish between the germs of the various orders of animal forms by microscopical or chemical analysis; but this need not induce us to assert that this can never be done.

Natural philosophers uniformly assert the impossibility of our ever being able to demonstrate the size of the atom. I regard it as hardly safe to longer iterate this saying; for the distance between these small bodies called centers and origins of motion (viz, the atoms) has been proved to be near the two hundredth millionth (200,000,000th) of an inch by late discoveries in the wave lengths of different color rays.

One of the most important of analogies is one that holds good in all forms of individual body, be it large or small, viz., "room" in which to work, sometimes called atmosphere.

The simplest motion is breathing—afflux and efflux, This lays the foundation of change, and is the initial of function in suns, planets and inhabitants of planets.

The deciduous character of the terræ, floræ and funæ of planets, and of the tissues of our bodies, are but the truly physiological processes by which the planets and our bodies come to their maturity, so as in turn to enrich the soil of the system to which each belongs, and thus take part in the completing of higher and ever higher career in the never ending line of progress of a divinely appointed endowment of power.

As deep of solar fullness calleth unto deep of planetary void, in the vast macrocosm of cosmical tissues, so calleth the atomic, molecular and psychic deeps of sense of deficiency to the deeps of pabulum of proteinaceous seas, and the deep of the sea of knowledge, to be infilled with the influx that satisfies by repletion of tissual, organic and psychic supply.

The blood corpuscles are the seed bodies of the various tissues, and the fluid blood is the food of the elements of the tissues, whereon they are sustained and enabled to do the work of organic and systemic career, as they take the place of the worn-out corpuscles and cells by pushing them to the surfaces to be shed as secretions, excretions or dejections unfit for further systemic use.

The duration or length of systemic career depends upon the condition of the organs that constitute the system.

And the term of continuance of the organs, in working condition, depends upon the supply of the tissues that together constitute the organs; and the renewal of the tissues also depends upon a supply of corpuscles (cells) that together form the filaments, fibers and webs of tissues of the various kinds by what has been called alternation of generation of these elemental bodies.

Thus it is evident that communion is the basis and formative act of society of all kinds and degrees, by which cosmical, planetary and subordinate systems are formed and maintained.

It will be thought to be "far-fetched," "misty," "cloudy" and "irrelevant" by many, if the attempt be made to name the process by which suns are produced, and to indicate the analogy that is the key to all function, in the result, on a cosmical scale, of the single law of gravitation.

Nevertheless, as heretofore, I take the risk, with only one request as condition, on the part of those who object, viz, that they be logical enough to wait until the statement is made and taken into account, before deciding for or against it.

To get at the analogy at the nearest point to us of the cosmical systems among which this earth holds its course, let us state a certitude as if it were a hypothetical proposition, and we may be the better able to catch the significance of analogies. The moon is nearing the earth. When it comes in full contact with the earth its mass-motion will be converted into molecular-motion, which will fuse both masses of moon and earth, and disrupt molecular affinities, setting them free to reduce the conjoined mass to a sun.

The function of *sun* is radiation, or giving. The function of planetary *void* is asking to be infilled—(demand).

How many moons the earth has had, and how many times it has been partly or wholly reduced to a state of sun, remains to be read by the records safely laid up in its crust; just as the scars in our bodies reveal the traumatic and functional lesions that have in succession held the dominion of nutrition in our bodies and marred the regularity of the arrangement of the elemental bodies composing the *scar-tissue* that will reduce them to earth-mould again so soon as it (scar-tissue) attains the balance of force in the systemic economy.

As habitable planets are produced by cooling down of suns through atomic satisfactions of *desire*, in the formation of ethers, gases, vapors, waters, colloids and solids; just so do satisfactions of molecular affinities produce the varied tissues whose concurrent gyres supply the organic and systemic functions, whose summations produce psychic or affectional and intellectual function in harmonial expression. And thus the planets become peopled with the various inhabitants in orders, classes, kingdoms, genera, species and varieties, in obedience to intelligent desire resident in elements seeking union.

Thus degrees of desire are the measure of the degrees of nearness of elemental bodies that constitutes what is called satisfaction of affinity. Therefore nearness and distance determine the quality of body. Thinnest, softest body is spirit body. Thickest, hardest, densest body is natural body. Therefore body, to be body at all, must have not only some degree of thinness, tenuity, but also of thickness, staticalness, stillness; and we therefore have to invoke force and form as the necessary elements of body, be it largest or least.

The difference, then, of that which occupies the interstellar spaces and our atmosphere is but a difference in degree of tenuity, and not in quality of matter. Hence all systems float in an identical ocean of substance, in which they subsist by degrees of teleological (distant), medioxumous (middle), and terraqueous (earthy) tensions of desire.

The *absolute* being incomprehensible to the finite mind, we can only deal with *relative* origins and careers of being and process.

Mind, being the exercise of consciousness is seriated perceptions, involves potential and active modes which constitute the seriating, or the beginning and completing, or finishing, of the special putting on and taking off of the tensions that comprise the mental acts.

A perfect anological understanding of any one of the seven aspects of endowment of the power to *unite*, from gravitation to spirit-power, involves knowledge of the whole domain of histology in the demand and supply of factors of function, in concurrent and divergent careers; which we now proceed to formulate in the order of their occurrence increative and procreative procession.

Confluence of consciousness and physics produces:

1. Confluent tissue, in nerve-mass, which subsequently differentiates into vesicular and fibrous forms of neural tissue.

2. Limitory tissue, in elastic, non-elastic and mixed forms of fiber.

3. Motory tissue, in voluntary, involuntary and mixed examples of muscles.

4. Secretory tissue (or glands), in lymphatic, conglobate and conglomerate forms. Ending in

5. The production of Statical tissue, in shells, bones and teeth; the last made up of three forms in cement, dentine and enamel, to meet the demand of statical requirement in completest degree in the last, which is composed of prismatic rods or crystals.

The offspring of confluence of atoms are molecules, the offspring of molecules are granules, and confluence of these produces corpuscles and cells, which are the elements of tissues, out of which the statical, limitory, motory, secretory and confluent varieties of tissues are formed, under the guidance of the differential types of tissues is: 1, Bones; 2, Fibrous or connective tissues; 3, Muscles; 4, Glands; and 5, Nerves, as the basis of a *mentory* apparatus which is the highest example of congeries of nerves or confluent tissues at present known. In this refinement of functioning body, automatic and instinctive intelligence becomes conscious intelligence, whereby histology and microscopy, and every other example of differential science becomes possible.

*Hunger* resides in the needy territories. The location of the *demand*, therefore, must be the seat of function.

Function is affected in the individual elements of the factors of function, which are called corpuscles or cells, according to our wont, habit, or school.

Hunger being appeased or satisfied, the demand abates or ceases.

The stomach is the grand *entrepot* of supplies for the whole system, and therefore for its own individual cellular necessities in its proper tissues, as well as for the rest of the organism, and only contributes its modicum of demand.

The mere filling of the stomach does not annihilate appetite, although it may appease, in a degree, the urgency of the sense of demand by mechanically distending the stomach, thereby forcing the blood-plasm within the intercellular territories more compactly against the needy bodies, thus favoring the appropriation of pabulum before out of the reach of the sphere of cell influence.

Upon the *intelligence*, then, of the cells do we find the satisfaction of hunger to depend, as certainly as that the formation of crystals depends upon chemical equivalency; which is but an example of *atomic intelligence* that knows when it is satisfied—saturated!

Crystallization, cellulation and corpusculation are the differential modes of absorption of radiance of sun-presence, and have their differential termini in the mineral, vegetable and animal kingdoms; all three of which are represented in the human system.

As protoplasm is, in first form, sea foam—*schleim*, as the Germans have it—it will be well for us to inquire the origin of slime of the sea—sea foam—meerschaum.

Bubbles of the sea are the result of five modes of motion or tension, viz: centripetal, centrifugal, orbital and revolving motions. and the absorption of sun radiance.

Centripetal motion is solar attraction.

Centrifugal motion is the tending of a moving body to go in a line.

Orbital motion is the resultant of these two; and Diurnal is revolving motion, or rotation on axis.

Sea foam arises out of the conflicts of the confluence of these currents of motion, in analogous, concurrent and competing presence at the junction of the atmosphere and the sea, or air and water. Protoplasm, or sea foam, takes its origin here, and is the basis of all tissue of rock, plant and animal. All belonging to rock or mineral is given to the domain of crystalosophy; that belonging to plants, to cellosophy, and that belonging to animal is remanded to the domain of corpusculosophy, which holds the values of the preceding two other forms of typal dominion. Therefore, in treating of corpusculation, cellulation and crystallization are understood, as possible change of the dominion, as well as that of solution and reduction to protaplasm again.

Cellulation: I have chosen this form as one least liable to the objections of novices who deem themselves able to assume the role of the expert. Investigators who are afraid of consequences, or are restive under conviction are not yet mature philosophers.

In using the term cell, to stand primarily for the least body capable of exercising vegetable or animal function, we void the conflicts coming out of the study of its origin, in this place, and shall have less cause for limping if we do not forget ourselves and allow or minds to wander from the line of alternations in the onward career of these primates of tissues, in their joint work of producing and maintaining organs and systems.

The differential function of cell is a complication of reception, transmission and absorption of force. This is the work of cell proper. And, as all forms of work wear the worker, (I will not say waste the worker, for this only occurs in doing needless work), the worker must be renewed or exchanged at each effort, so that career or continuity of work may be secured.

How may we determine cell career? I know of no other way to do this so satisfactorily as to watch the development of the various cells and products of cells in plants and animals as they do the work of their careers in the appropriation of pabulum, working and twisting into tissues, and successfully crowding their predecessors to the surfaces of organs, to be shed when no longer fit to constitute any part of an organ.

Secretion and excretion are examples of moulting of the elemental primates of tissues.

In the example of secretions, the peripheral cells are shed, in diffluent mass by solving entirely, and bodily, by solution of their points of attachment on all sides. When the shed corpuscles, solved, partly solved, and entire, are to serve other than lubricant uses, they constitute a secretion; when in excess, or effete, they constitute what is called excretions —both secretion and excretion being the example of end of career of corpuscles and cells.

After this succinct nomination and tracing of career of tissual elements from the first appearance of blood corpuscles to its exit in shed epithelium, it will be well to study the following tabulated series of modes of motion and metamorphosis of bodies and processes before applying ourselves to the grand retrospect, introspect and prospect that completes these mere headings of histology and miroscopy.

| | | | | | |
|---|---|---|---|---|---|
| VII. Spirit Power. | Glacial and Recent. (Cenozoic) | Seeding Plants. (Dicotyle-dones.) | Spiritual Sense. | Intuition. | Spiritual Love (Love of God and Heaven.) |
| VI. Vital Force. | Tertiary. (Mammals) | Fruiting Plants. (Exogens.) | Binocular Sight. (Mental Sight Reason.) | Reason. | Universal Love. |
| V. Chemism. | New Red Sandstone. (Lias, Weal-don, etc.) | Flowering Plants. (Monocotyle-dones.) (Endogens.) | Smell. | Reflection. | Filial Love. |
| IV. Electrism. | Carboniferous | Leaf Plants. (Ferns, spores on leaves.) | Hearing. | Imagination. | Fraternal Love. |
| III. Light. | Lower Fossili-ferous. (Silurian, Cambrian, etc.) | Tracheal Plants. (Branches.) | Monocular Sight. | Memory. | Parental Love. |
| II. Levitation. (Apartness.) (Expansion.) (Heat.) | Stratified. (Azoic.) | Duct Plants. (Trunk.) | Taste. | Conception. | Conjugal Love. |
| I. Gravitation. (Together-ness.) (Contraction.) (Cold.) Motism. (Modes of Motion.) | (Igneous. Amorphous.) Mineralism. (Attraction and Repulsion.) | Cell Plants. (Root.) Vegetism. (Botany.) | Touch. Animism. (Animality.) | Perception. Mentism. (Intellectual Faculties.) | Self Love. Emotism. (Affectonal,) |

| | |
|---|---|
| VII. | Pneumatogeny. (Emotism.) (Spirits.) |
| VI. | Psychogeny. (Mentism.) (Rational Natures.) |
| V. | Biogeny. (Curvilinear.) (Vegetism and Animism.) |
| IV. | Cosmogeny. (Rectangular.) (Mineralism.) |
| III. | Morphogeny. (Annulated.) (Offspring.) |
| II. | Pantogeny. (Motism—b.) (Pater.) |
| I. | Substance. (Motism—a.) (Mater.) |

### GRAND RETROSPECT, INTROSPECT AND PROSPECT OF BEING, BODY AND PROCESS.

When the desire to have associates went forth from the divine counsel in the heavens, and was expressed in the recorded "Let us make man," the foundations of society were firmly laid in the very inmost nature of the creature that was there and then pronounced "very good." If good then, and very good at that, where is the door or even hint of point of entrance for anything but good?

Absolute or abstract good is incomprehensible, therefore out of the question now. That which supplies a demand, fills a need, is the only good that can by possibility be so called. Therefore, that which is good to the needy, and satisfactory to his estimation, is indifferent or repulsive to another who is full and lacking nothing of which his sense takes cognizance.

To walk "together" we must be "agreed" (*i. e.*) in like or similar states and conditions of necessities in deficiency and sufficiency.

All those who deem themselves their own keepers, and able to take yokes upon their neckes that neither they nor their fathers were able to bear, have quite forgotten to ask to be led.

Under a sense of the truth of the Scripture that saith, "It is not in man that walketh to direct his steps," our helplessness is ever before us, and in our deepest consciousness we are sure that desire is before will (volition), and spontaneity before calculation.

Obedience that is not instant and spontaneous is but second rate at best, and brings but partial sense of satisfaction to the soul—the plane of the sense of need, or appetite.

Those who "serve" the divine from a "sense" of duty only blasphemously give remnants of their affections, and are trying (Simon-like) to buy their way to heaven. All such have but embryonic ideas of heaven, forgetting the saying, "the kingdom of God is within you."

Without opposites we have no differential, sharp apprehensions of state or place. Therefore both hell and heaven are involved in what is called happiness. A proper use of hell converts it into heaven, and an improper use of heaven transmutes it into hell.

Until serial order of the development of body and mind be formulated and taught, shall we ever come to our mental and moral majority, in which we are a law (Lord), unto ourselves in which our spontaneities ever concur with the behests of the law (Lord).

"Are there few that be saved?" Verily, few indeed attain to mental and moral childhood even, at first efforts at evolution of the complete being.

Childhood in the growth of the body is reckoned from the first to the seventh year, youth from the seventh to the fourteenth year, and adolescence from the fourteenth to the twenty-first year.

The moral and religious life has also a serial order of correspondence with this formula. But where are the living examples of the youth and adolescence of these categories? Echo answers, where?

In natural history the mineral kingdom has been regarded as non-living. The straight lines of this kingdom, and the possible forms of body producible by their intersections and conjuctions, are but statical forms devoid of active motion, therefore called dead forms.

All morals and religions based upon "authority" (external denomination) are but the mineralistic analogues of morals and religion, only useful as base or sub-stratum upon which to erect the *Paternity* and *Immanuelism* for the establishment of which they were produced, as stages in the growth of æsthetic society among men.

The "lording it over God's heritage" in religious circles governmental bodies, schools and scientific societies, is fast falling into the mere stages and periods of geological record of past denominations of forces now cancelled and carried into higher fields and broader realms of usefulness; although the incumbents of popedoms, priesthoods, and professional chairs take little cognizance of the departure of their energies.

If, then, "the fine gold has become dim, and the beautiful garments moth eaten," where are we to look for safety? Evidently not in tarrying where we are, nor in going backward; but in a ready acceptance of the beckonings of the heavens that hold out kindly hands to charm us out of the necessary hells of development into the embrace of higher, purer, diviner degrees of acquiescence in the love and wisdom that energizes and pacifies—"satisfies every living thing."

Not in conservatism, not in retrogression, not in head religion and formulæ, however fascinating to the senses, shall we find our account, but in ready obedience even unto death, with the ardent exclamation of, "As it is written in the volume of thy book, lo! I come to do thy will, O God!" And thus, without hesitation, and with the sweet ecstacy of divine abandonment, rush to the fulfillment of every requirement of light and life, of law and love, reckless of results by the very ardor begotten of obedience, and, dancing on the foam of the breaking crest of the advance wave of real progress, catch and appropriate the divine afflatus of Perpetual Adolescence—the birthright and inheritance of the sons and daughters of the Almighty!

